# Sensa Mobile App for Managing Stress, Anxiety, and Depression Symptoms: Pilot Cohort Study

**DOI:** 10.2196/40671

**Published:** 2023-04-13

**Authors:** Sarunas Valinskas, Marius Nakrys, Kasparas Aleknavicius, Justinas Jonusas

**Affiliations:** 1 KiloHealth Vilnius Lithuania; 2 Lithuania Business University of Applied Sciences Klaipėda Lithuania

**Keywords:** depression, anxiety, stress, depressive, DASS-21, mobile application, CBT, cognitive behavioral therapy, psychotherapy, mHealth, mobile health, Sensa, app, application, health care, intervention, effectiveness, assessment, symptoms, treatment, mental health

## Abstract

**Background:**

An increase in depression, anxiety, and stress symptoms worldwide, attributed to the COVID-19 pandemic, has been reported. If not treated, it may negatively affect a person's everyday life by altering physical and social well-being and productivity and increasing expenditure on health care. Cognitive behavioral therapy (CBT)–based interventions are gaining popularity as a means to reduce stress and alleviate anxiety and depression symptoms. Moreover, CBT delivered through a mobile app has the same elements as traditional CBT training (eg, guided discovery). However, unlike conventional training, users of mobile apps are allowed to tailor their own experience at their own speed and schedule.

**Objective:**

This study aims to analyze Sensa users’ retrospective data and explore the dose-duration effect to find the optimal usage time when the user showed results.

**Methods:**

The study cohort comprised 381 consecutive community-based nonclinical users who started using Sensa between October 2021 and March 2022. All users included in the study took the Depression Anxiety Stress Scale-21 (DASS-21) assessment at least 2 times. Other parameters from the database containing all self-reported data were gender, number of active days, total time of use, and age. The primary outcome of the study was a change in the DASS-21 score. Statistical analyses were performed using GraphPad Prism (version 9, GraphPad Software). In addition, a logistic regression model was created to predict how the obtained independent parameters influenced the DASS-21 score.

**Results:**

The main finding of our study was that the majority of participants who started using Sensa were experiencing depression, anxiety, and stress symptoms (92.13%, 80.05%, and 87.93%, respectively). There was a statistically significant decrease of the DASS-21 subdomain scores after the use of the application (anxiety: mean 7.25, SD 4.03 vs mean 6.12, SD 4.00; *P*=.001; depression: mean 11.05, SD 4.26 vs mean 9.01, SD 4.77; *P*=.001; stress: mean 11.42, SD 3.44 vs mean 9.96, SD 3.65; *P*<.001). Finally, the logistic regression model showed that users who were using the app for more than 24 days and had at least 12 active days during that time had 3.463 (95% CI 1.142-11.93) and 2.644 (95% CI 1.024-7.127) times higher chances to reduce their DASS-21 subdomain scores of depression and anxiety, respectively.

**Conclusions:**

Using the Sensa mobile app was related to decreased depression, anxiety, and stress symptoms.

## Introduction

An increase in depression, anxiety, and stress symptoms worldwide, attributed to the COVID-19 pandemic, has been reported in the literature [1-6]. The high prevalence of depression, affecting up to 17% of the population, is well documented [7]. Depression episodes are strongly related to suicidal behavior that increases the risk of suicide, with an economic cost of more than US $58 billion annually [8,9]. Additionally, earlier data report that more than 30% of US adults experience some type of anxiety disorder at some point in their lives [10]. A recent systematic review showed similar results, reporting the prevalence of anxiety to be 31.9% (95% CI 27.5-36.7) [11]. However, during the COVID-19 outbreak, the prevalence of anxiety increased primarily among patients with COVID-19 and reached 47% (95% CI 37.0-57.0) [12]. Finally, the reported prevalence of stress among the general population is 29.6% (95% CI 24.3%-35.4%), while the prevalence among health care specialists is much higher (45%, 95% CI 24.3%-67.5%) [11,13].

If not treated, depression, anxiety, and stress may negatively affect a person’s everyday life by altering physical and social well-being and productivity, and increasing the general expenditure on health care because of the increased prevalence of chronic diseases [14-16]. Unfortunately, a treatment gap results in a situation when people with depression and other mental illnesses remain untreated [17]. Qin and Hsieh [18] stipulate that 4 obstacles preventing people from accessing timely treatment when needed are stigma, costs, geographic maldistribution of health services, and low-quality health care services.

Cognitive behavioral therapy (CBT)–based interventions are gaining popularity as a means to reduce stress and alleviate anxiety and depression symptoms [19-21]. Hofmann et al [22] state that mental health and behavioral problems may be decreased by reducing dysfunctional cognitions through cognitive techniques such as restructuring, relaxation, meditation, and others. However, the need for an experienced practitioner and the time and resources needed to prepare one slows down the widespread implementation of this method in general practice [23].

Interventions built upon CBT principles and delivered through a mobile app have the same elements of traditional CBT training (eg, guided discovery). However, unlike conventional training, users of mobile apps are allowed to tailor their experience at their own speed and schedule [24]. Furthermore, considering that over 80% of the US adult population have a smartphone [25], a mobile app–based approach to implementing CBT-based techniques becomes an attractive option that can even provide real-time assessments of the users’ mental health. It has been shown in several studies that mobile app–based CBT interventions have the potential to overcome the majority of burdens affecting timely access to mental health care services and can be an effective tool for people with depression, stress, and anxiety [26-31]. However, the efficacy of mobile app–based CBT interventions highly depends on user retention and engagement [32]. Nevertheless, a recent meta-analysis by Linardon et al [33] summarizes that even though mobile app–based mental health interventions are not intended to replace face-to-face consultations, they provide a cost-effective and highly accessible intervention for those in need.

One of the apps commercially available in the mobile app stores is called Sensa (Kilo Health). This app was created upon the principles of CBT. By implementing these techniques, the app aims to help users mitigate mental health issues such as anxiety, stress, depression, and others.

This study aimed to analyze the retrospective data of Sensa users and explore the dose-duration effect of the app in question to find the optimal usage time when users showed results.

## Methods

### Study Design and Recruitment

This was a retrospective chart review study. The study cohort comprised 381 consecutive community-based users who started using Sensa between October 2021 and March 2022 and answered the Depression Anxiety Stress Scale-21 (DASS-21) questionnaire at least 2 times. There was no user screening process, so the sample may have included clinical and nonclinical users. The first assessment was made during the account setup stage, and the rest of the assessments were made during app usage time. The users were prompted once per week to complete the DASS-21 assessment. A total of 78 (19.5%) users were male and 303 (81.5%) were female (*P*=.001).

Sensa is available for download from the App Store and the Google Play store. Users are able to register an account within the app or on the Sensa website. During account registration, the users enter their name, age, gender, and email address and are asked how they currently feel in general (Terrible/Bad/Okay/Good/Great). After downloading the app, the users receive an onboarding email that explains how to navigate in the app and gives some initial directions to the in-app onboarding (eg, open Sensa, go to “My Plan,” track your mood, explore in-app exercises) and explains the importance of following the plan and doing the exercises. The onboarding is designed to explain and show the app’s features and how to navigate between them. The users can choose to complete a DASS-21 assessment at any time, but they have to wait at least 7 days after completing it for it to become available again. If a user has an open assessment that is not yet completed, they receive a reminder push notification from the app every 7 days. This study did not include users who did not complete the DASS-21 assessment during account setup.

### Intervention

**Figure 1:**
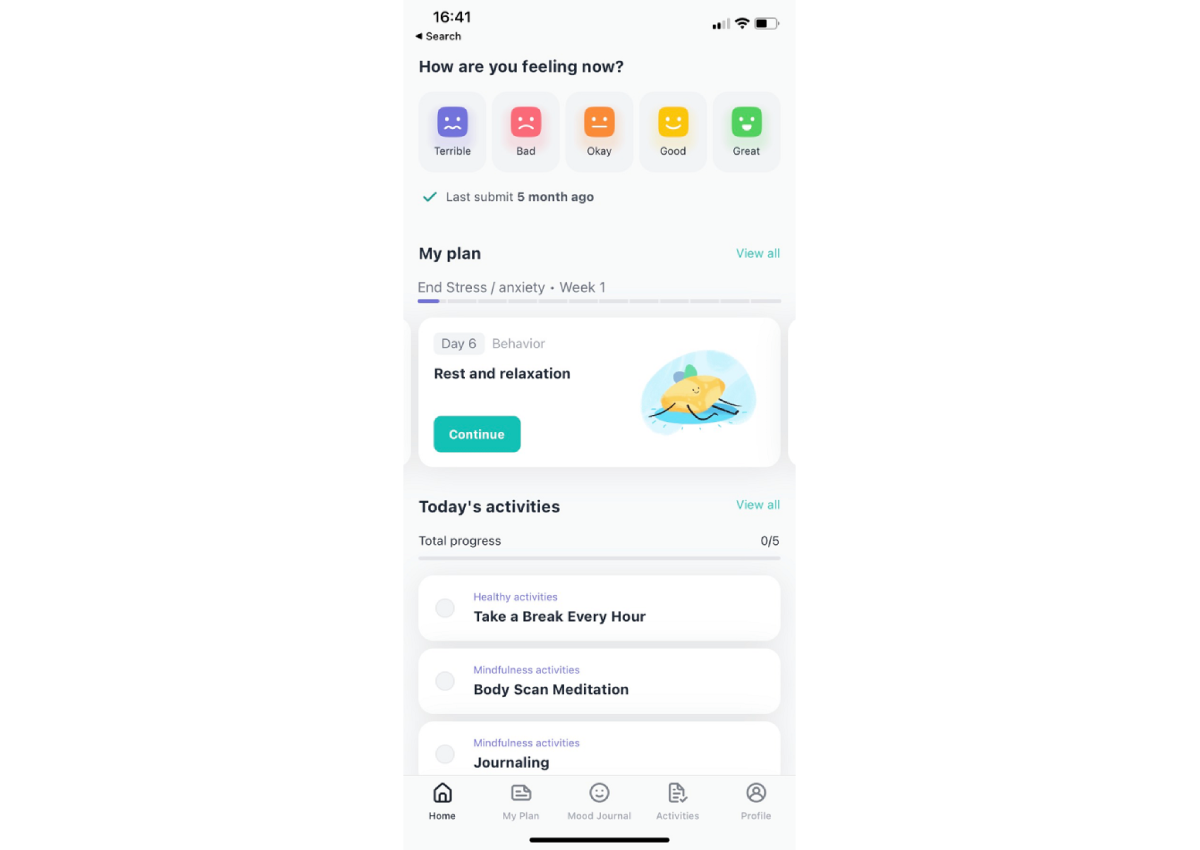
Sensa home screen with summaries of user plans and daily activities.

**Figure 2:**
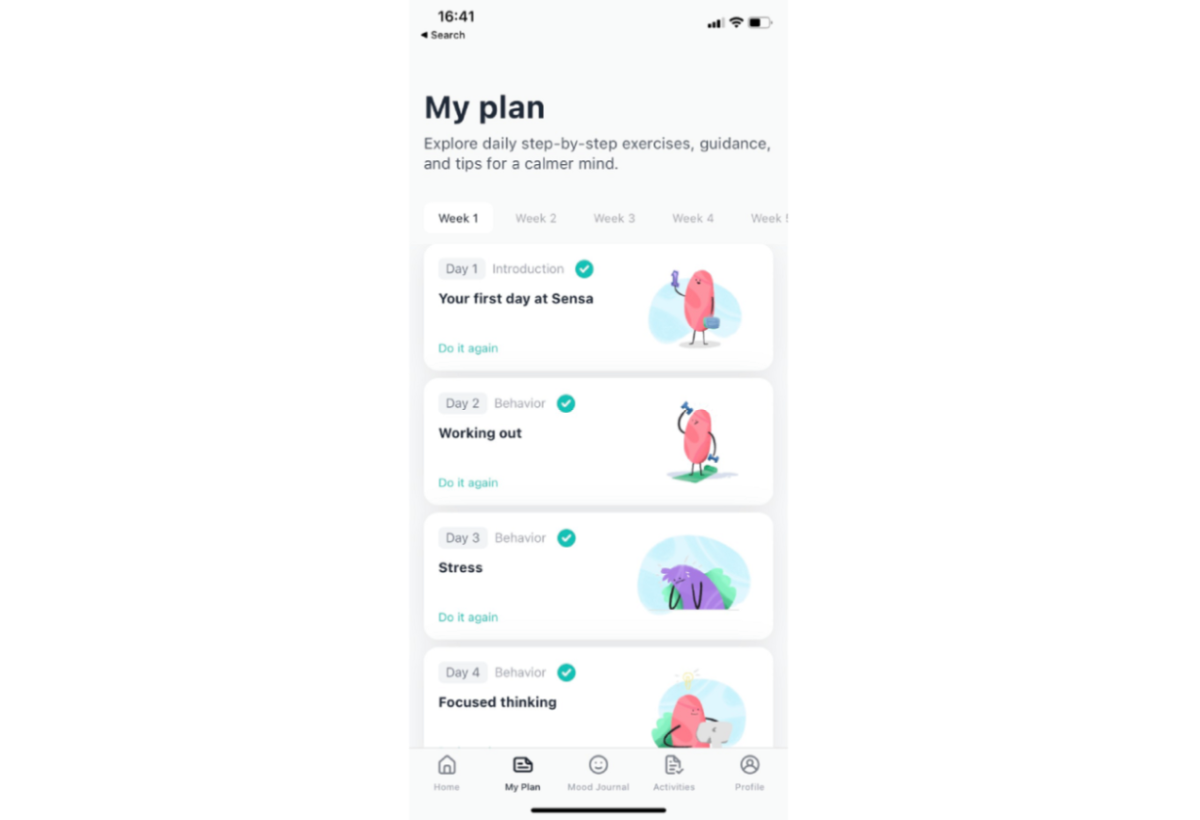
The “Activities” tab with a summary of each day’s activities and possibilities to manage existing and add additional activities.

**Figure 3:**
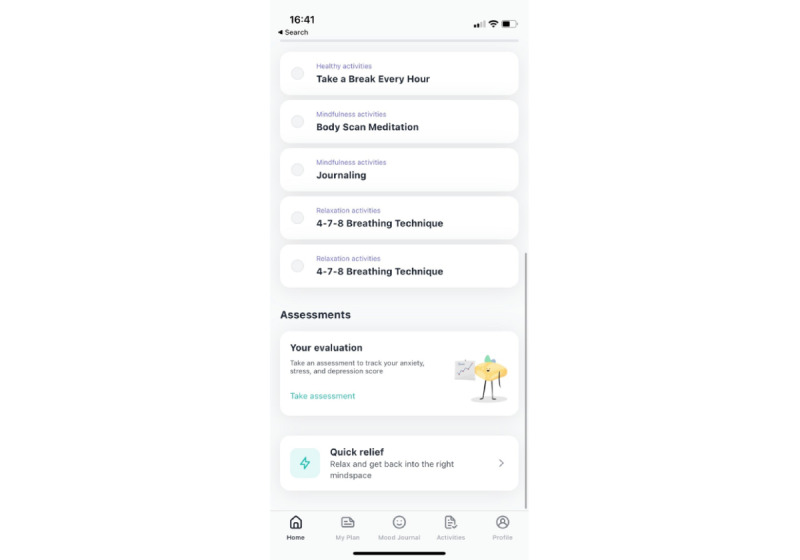
Continuation of the home screen with available assessments and quick-relief exercises.

Sensa provides its users with a CBT-based intervention that incorporates acceptance and commitment, dialectical, and schema therapies and was designed by licensed psychotherapists specializing in CBT. The Sensa mobile app uses proven CBT techniques. Daily lessons and readings about mental health and how thoughts and emotions are connected to behavior help users terminate negative behavior patterns. All this is achieved by completing daily tasks, which take up to 30 minutes to complete throughout the daytime. Completing these daily tasks creates habits that help build resilience toward stress, anxiety, or depression as users acquire knowledge on bringing back mental stability. Additionally, the app has a mood journal. The users observe their emotions and write them down in the app, which helps them see and accept what kind of situations trigger those emotions. Observing and recognizing one’s emotions is based on research showing that effort toward accepting instead of judging one’s experiences may help one feel less negative emotion and thus achieve better psychological health [34]. The app also asks about thoughts, allowing users to understand that not situations but their interpretations of them trigger their emotional responses. For this, a relevant strategy is a cognitive reappraisal, which allows one to reinterpret the meaning of a stimulus to change the emotional response to it. Research has demonstrated that it may be related to anxiety symptom improvement [35]. Finally, if the user needs help on the spot, various quick relief exercises such as deep breathing, grounding, and other proven practices are provided. See Figures 1-3 for reference screenshots of Sensa.

### Measures

DASS-21 was used in this study to measure psychological distress [36]. The users rated their experience of symptoms of depression, anxiety, and stress over the previous week on a 4-point scale, ranging from 0 (did not apply to me at all) to 3 (applied to me very much or most of the time). Items in each subscale were summed to provide scores for depression, anxiety, and stress symptoms, with higher scores indicating greater severity of symptoms. The scoring guide has been published by the Psychology Foundation of Australia [37].

The number of active days (AD) was obtained from the application’s login data. The sum of days when the user logged into the app was determined as AD. Total time of use (TT) was calculated by subtracting the first login date from the last. Gender, age, and starting DASS-21 score values were self-reported by users during the account setup stage. For the final DASS-21 score values, the user’s last DASS-21 assessment was used.

### Statistical Analyses

GraphPad Prism (version 9, GraphPad Software) was used for the statistical analysis of the obtained data. The normality of continuous variables was determined using the Shapiro-Wilk test. The Wilcoxon signed rank test was used to analyze the paired samples when related nonnormally distributed samples were compared. One-way ANOVA was used for multiple comparisons. The value of significance was chosen to be .05.

A logistic regression model was created when statistically significant differences between starting and final DASS-21 mean scores were found. The difference between the DASS-21 scores was chosen as the dependent parameter. DASS-21 scores were coded in the following manner: users who experienced a decrease in the DASS-21 score from extremely severe, severe, moderate, or mild to normal were coded as 1, while other users were coded as 0. The covariates were exposure level (low TT–low AD, low TT–high AD, high TT–low AD, and high TT–high AD). Low and high exposure was determined according to the median of the corresponding value. If it was lower than the cohort’s median, it was considered to have a low (and vice versa) DASS-21 assessment count and days between DASS-21 assessments. The logistic regression model was created to analyze how independent parameters (covariates) influenced the dependent parameter.

### Ethical Considerations

The investigation was performed in accordance with the ethical standards of the institutional review board (this retrospective chart review study was approved by the Biomedical Research Alliance of New York [BRANY] in June 2022; registration: 22-08-438-939) and with the 1964 Helsinki Declaration and its later amendments or comparable ethical standards. As this is a minimal-risk retrospective chart review study, it was waivered by the institutional review board from obtaining informed consent from the users whose data were included in the study. Additionally, all data included in the study were deidentified, and the authors could not retrace the identities of the users included in the study. Finally, there was no compensation paid to the participants.

## Results

The majority of the participants experienced depression, anxiety, and stress symptoms when they started using Sensa (Table 1). After the Wilcoxon signed rank test was performed, a statistically significant decrease in self-reported DASS-21 scores was found among all users (Table 2).

User stratification according to exposure level showed that more prolonged exposure to the app was associated with a statistically significant reduction in DASS-21 scores (Table 3). It was found that all groups except the low exposure group (low TT–low AD) managed to decrease DASS-21 anxiety, depression, and stress scores (*P*<.05). Additionally, multiple comparisons of DASS-21 depression score changes between exposure groups showed a statistically significant difference for different levels of exposure groups (F_3,377_=3.158; *P*=.02). Tukey post hoc analysis showed that the “high TT–high AD” group statistically significantly decreased the DASS-21 depression score when compared to that of the “low TT–low AD” group (*P*=.02). Multiple comparisons of DASS-21 anxiety and stress score changes between exposure groups showed no statistically significant differences.

The creation of the logistic regression model followed. The parameters used in the model coded changes of DASS-21 scores as the dependent variable and exposure level, DASS-21 assessment count, and average time between assessments as the independent variables (Table 4). The logistic regression model showed that only long and frequent app use resulted in a statistically significant difference in the DASS-21 depression and anxiety scores from extremely severe, severe, moderate, and mild to normal. In addition, users who were in the high-engagement group (high TT and high AD) had a greater chance of reducing their depression and anxiety scores when compared to that in the low-engagement (low TT and low AD) group (odds ratio 3.463, 95% CI 1.142-11.93; odds ratio 2.644, 95% CI 1.024-7.127, respectively). Moreover, the logistic regression model showed that an increased time interval between DASS-21 assessments lowered the chance of reducing the DASS-21 stress score.

**Table 1:** Descriptive data of the final cohort. Depression Anxiety Stress Scale-21 (DASS-21) scores are shown as means with SDs. Active days of use and the total time of use are shown as the median in days with ranges.

Characteristic				Value		*P* value
The time between DASS-21 assessments, mean (SD)				12.22 (6.24)		N/A^a^
Active days of use (days), median (range)				12 (2-52)		N/A
Total time of use (days), median (range)				24 (5-53)		N/A
**DASS-21 scoring, n (%)**						
	**DASS-21 depression**					.001
		Normal	30 (8)			
		Mild	33 (9)			
		Moderate	95 (25)			
		Severe	120 (31)			
		Extremely severe	103 (27)			
	**DASS-21 anxiety, n (%)**					.001
		Normal	76 (20)			
		Mild	70 (18)			
		Moderate	53 (14)			
		Severe	71 (19)			
		Extremely severe	111 (29)			
	**DASS-21 stress, n (%)**					.001
		Normal	46 (12)			
		Mild	61 (16)			
		Moderate	125 (33)			
		Severe	126 (33)			
		Extremely severe	23 (6)			
	**First DASS-21 assessment score, mean (SD)**					.001
		DASS-21 depression	11.05 (4.26)			
		DASS-21 anxiety	7.25 (4.03)			
		DASS-21 stress	11.42 (3.44)			

^a^N/A: not applicable.

**Table 2:** Comparison of starting and final means of Depression Anxiety Stress Scale-21 (DASS-21) depression, anxiety, and stress scores (values are given as means with SD).

DASS-21 subscore	Starting score, mean (SD)	Final score, mean (SD)	*P* value
Anxiety	7.25 (4.03)	6.12 (4.00)	.001
Depression	11.05 (4.26)	9.01 (4.77)	.001
Stress	11.42 (3.44)	9.96 (3.65)	.001

**Table 3:** Stratification of users into groups according to their exposure level.

Characteristics		Group 1^a^ (N=84)	Group 2^b^ (N=100)	Group 3^c^ (N=99)	Group 4^d^ (N=98)
**Anxiety, mean (SD)**					
	Starting	7.1 (3.81)	7.39 (4.07)	7.09 (4.16)	7.42 (4.08)
	Final	6.93 (3.9)	6.11 (3.97)	5.87 (3.97)	5.69 (4.05)
	Difference	−0.17 (3.93)	−1.28 (3.49)^e^	−1.22 (3.87)^e^	−1.72 (3.88)^e^
**Depression, mean (SD)**					
	Starting	10.69 (4.39)	11.76 (4.29)	10.46 (4.23)	11.21 (4.11)
	Final	9.83 (4.46)	9.39 (5.03)	8.49 (4.46)	8.46 (4.98)
	Difference	−0.86 (4.22)	−2.37 (3.73)^e^	−1.97 (4.01)^e^	−2.76 (4.91)^e^
**Stress, mean (SD)**					
	Starting	11.08 (3.69)	12.00 (3.23)	11.19 (3.54)	11.36 (3.26)
	Final	10.56 (3.71)	10.32 (3.58)	9.55 (3.59)	9.5 (3.65)
	Difference	−0.52 (3.79)	−1.68 (2.77)^e^	−1.63 (3.43)^e^	−1.85 (3.39)^e^

^a^Group 1: low total time of use (TT)–low number of active days (AD).

^b^Group 2: low TT–high AD.

^c^Group 3: high TT–low AD.

^d^Group 4: high TT–high AD.

^e^Statistically significant difference, with a *P* value less than .001.

**Table 4:** Logistic regression model predicting the likelihood of reducing the Depression Anxiety Stress Scale-21 (DASS-21 depression, anxiety, and stress scores from extremely severe, severe, moderate, and mild to normal.^a^

Variable			Coefficient (SE)		*P* value	Odds ratio (95% CI)
**Depression**						
	Variable 1^b^	0.806 (0.549)		.14		2.240 (0.804-7.243)
	Variable 2^c^	0.853 (0.579)		.14		2.347 (0.784-7.953)
	*Variable 3* ^d,e^	*1.242 (0.588)*		*.03*		*3.463 (1.142-11.93)*
	Variable 4^f^	0.284 (0.179)		.11		1.329 (0.932-1.896)
	Variable 5^g^	0.001 (0.027)		.96		1.001 (0.945-1.053)
**Anxiety**						
	Variable 1	0.637 (0.435)		.14		1.892 (0.825-4.633)
	Variable 2	0.958 (0.456)		.48		2.608 (1.089-6.626)
	*Variable 3*	*0.972 (0.490)*		*.04*		*2.644 (1.024-7.127)*
	Variable 4	0.096 (0.172)		.58		1.101 (0.778-1.542)
	Variable 5	−0.027 (0.172)		.28		0.972 (0.921-1.020)
**Stress**						
	Variable 1	−0.329 (0.406)		.42		0.719 (0.321-1.602)
	Variable 2	0.122 (0.431)		.78		1,130 (0.480-2.637)
	Variable 3	0.657 (0.447)		.14		1.930 (0.801-4.675)
	Variable 4	−0.001 (0.175)		>.99		0.999 (0.701-1.407)
	*Variable 5*	−*0.085 (0.035)*		*.02*		*0.918 (0.851-0.977)*

^a^The model was created using the data of Sensa users with 2 or more DASS-21 assessments. The general model showed that only long and frequent exposure (high TT–high AD) was associated with decreasing DASS-21 anxiety and depression scores.

^b^Variable 1: low total of time of use (TT)–low number of active days (AD) versus low TT–high AD.

^c^Variable 2: low TT–low AD versus high TT–low AD.

^d^Variable 3: low TT–low AD versus high TT–high AD.

^e^Statistically significant scores are italicized.

^f^Variable 4: DASS-21 assessment count.

^g^Variable 5: average days between DASS-21 assessments.

## Discussion

### Principal Findings

The main finding of our study was that there was a statistically significant difference between starting and final DASS-21 subdomain scores. Secondary findings were that prolonged exposure to the app was associated with a statistically significant reduction in DASS-21 subdomain scores. Finally, the logistic regression model showed that users who were using the app for more than 24 days and had at least 12 active days during that time had 3.463 (95% CI 1.142-11.93) and 2.644 (95% CI 1.024-7.127) times higher chances to reduce their DASS-21 subdomain scores of depression and anxiety.

There is mounting evidence of successful usage of CBT- and mindfulness-based mobile interventions to alleviate symptoms of depression, anxiety, and stress during the prepandemic time [26-31]. However, fewer studies analyzed the efficiency of such mobile interventions during the COVID-19 outbreak. A study by Kubo et al [38] analyzing female participants with expressed symptoms of depression showed improvements in depression and perceived stress symptoms (mean changes in Patient Health Questionnaire [PHQ-9] and Perceived Stress Scale scores were −6.0, SD 5.5 and −5.6, SD 7.3, respectively, with *P*<.05) while using the Headspace mobile app (a mobile app that provides mindfulness-based self-paced meditation). Similar results have been shown using the Calm app (a mindfulness-based meditation and relaxation app) [39]. The authors report that the intervention group reduced perceived stress by 4.28 (95% CI 1.68-6.88; *P*=.002) during the app usage period. Furthermore, a longitudinal waitlist trial by Song et al [40] demonstrated that a mobile app called CMSC statistically significantly reduced anxiety and insomnia symptoms evaluated by the PHQ-9 and attributed to the COVID-19 pandemic using a CBT approach. Finally, a randomized controlled study by Liu et al [41] reported that CBT-based computer programs used concurrently with the usual treatment statistically significantly demonstrated a greater reduction of depression and anxiety symptoms in comparison with that of standard treatment only (measure tools and differences are shown accordingly^:^ Hamilton Depression Rating Scale −7.01, 95% CI −7.90 to −6.12; Hamilton Anxiety Scale −5.84, 95% CI −6.69 to −4.99; Self-Rating Depression Scale −12.92, 95% CI −14.70 to −11.14; Self-Rating Anxiety Scale −14.16, 95% CI −16.00 to −12.33).

As measured by DASS-21, our results showed a trend similar to the abovementioned studies that CBT-based mobile app use may be associated with reduced symptoms. Those using the app statistically significantly lowered their depression, anxiety, and stress symptoms (Table 2). Moreover, when users were stratified by their exposure to the app, it was found that users in the low-engagement group did not reduce their DASS-21 score in any subgroup (Table 3). Interestingly, in the logistic regression analysis, the average number of days between DASS-21 assessments was related to the probability of increasing perceived stress symptoms. However, those using the app for more than 24 days and having at least 12 active days during usage time had more than 3 times higher chances of reducing their DASS-21 subdomain scores of depression and anxiety than those of less active users (Table 4).

Comparing these results to those of the studies mentioned above is not straightforward. First, many apps that were mentioned are mindfulness based. While mindfulness and CBT differ in their approach, they share some key principles [42]. Both approaches emphasize the importance of awareness and acceptance of one’s thoughts and feelings. In CBT, this is achieved through identifying and challenging negative thoughts, while in mindfulness, it is achieved through nonjudgmental observation of thoughts and feelings. Thus, these 2 approaches are often combined in interventions to promote mental well-being. Additionally, the abovementioned studies were done in the COVID-19 setting, which may have increased the measured baseline levels of symptoms and affected the effect size of the interventions. They also targeted specific populations (eg, students and patients with COVID-19), whereas the Sensa user sample was not controlled. In that sense, our results showed an association between Sensa use and reduced DASS-21 subdomain scores in an uncontrolled real-world setting, where users can access a publicly available app but drop out any time, potentially giving insight into how the app users may behave outside of studies.

This study has several limitations that must be mentioned. First, this is a retrospective chart review study, and no control group was established. Having a control group would allow us to more directly estimate Sensa’s true effect and make more robust conclusions, which cannot be done now. However, retrospective chart review studies can still have value. They lay the background foundation for future randomized controlled trials, raise new hypotheses, and in this particular case, retrospective results bring a small but significant portion of evidence to the digital mental health data pool. Second, the user cohort was widely heterogeneous because data from all consecutive users were used in the analysis. A heterogeneous sample has large differences among its data points, so the relationships between the predictors (engagement) and target variable (DASS-21 subdomain scores) can be inconsistent and difficult to predict. This lowered the accuracy of the created logistic regression model. To mitigate this, future research should consider using a larger sample and stratified sampling to lower the sample’s heterogeneity, making logistic regression models more accurate. Third, the study did not control for random effects. As this was a real-world study, user criteria and environment were not strongly controlled, so multiple unknown variables may have affected our observed outcomes. Additionally, this is a retrospective chart review study, and data of all consecutive users who had at least 2 DASS-21 measures, without control of time between the measures, were added to the data set, which is a notable limitation to what conclusions can be drawn about Sensa. Some users may have completed the assessments a week apart, while others with a much longer gap. A median-split methodology was also used for our variables of engagement, which may have introduced additional random errors. A median split allowed us to easily create user engagement categories, but it did not capture within-group variation properly. For example, users both slightly and very much above the median were both assigned to one group even if the difference between their engagement values was larger than when compared to those just below the median, creating groups that were more different than alike within. Another sample may have yielded different results with this method, although our sample size may have helped alleviate this effect. Future research could consider using more robust methodologies, for example, growth curve modeling, to compare the outcomes of more and less engaged users defined (eg, in quartiles) with fixed assessment time points. This would capture and show the effect of engagement more clearly and address the errors introduced by differing assessment time points. Finally, data about the general health status of the users, their comorbidities, the medicines they were using, and their adherence to them were not collected. This study also did not collect user feedback and satisfaction data, which may be desirable in the future for similar studies. Thus, conclusions about Sensa’s effect on its users must be drawn with caution. Statistically significant tendencies were noticed, but in light of the aforementioned limitations, they cannot be attributed to the effect of Sensa. Additionally, more controlled studies are still required for Sensa and are planned in the near future.

### Conclusions

This study reports the retrospective outcomes of the Sensa mobile app. It was found that most participants were experiencing depression, anxiety, and stress symptoms. The usage of the Sensa mobile app was related to a decrease in these symptoms. Additionally, active and longer usage of the app greatly increased the odds of reducing the symptoms of depression and anxiety. Further prospective studies are needed to investigate the effects of CBT interventions delivered via the Sensa mobile app.

## Abbreviations

AD: number of active days
CBT: cognitive behavioral therapy
DASS-21: Depression Anxiety Stress Scale-21
PHQ-9: Patient Health Questionnaire
TT: total time of use

## Data Availability

The data sets generated and analyzed during the current study are not publicly available due to the protection of confidential information of Kilo Health business and Kilo Health users but are available from the corresponding author upon reasonable request.
